# Federated Learning Based on Fuzzy Fusion Rules for Chemical Production Process Fault Diagnosis

**DOI:** 10.3390/s26113545

**Published:** 2026-06-03

**Authors:** Yuting Xu, Wangzhuo Yang, Shuwang Du, Meifu Zhang

**Affiliations:** 1 Department of Automation, Zhejiang University of Technology, Hangzhou 310023, China; xuyuting2026p@163.com (Y.X.); yangwz@zjut.edu.cn (W.Y.); 2 Joint Research and Development Center of Zhejiang University of Technology and Beijing United Information Technology Co., Ltd., Hangzhou 310023, China; zhangmeifu@ueiibi.com; 3 Beijing United Information Technology Co., Ltd., Beijing 100070, China

**Keywords:** personalized federated learning, fuzzy rules, fault diagnosis, multi-source fusion

## Abstract

Process data plays a vital role in diagnosing fault sources in chemical production. However, such data contain rich process information and are often sensitive, making direct analysis infeasible due to privacy concerns. Although federated learning mitigates data leakage risks, the conventional averaging strategy falls short in achieving high fault identification accuracy, especially under non-independent and identically distributed (non-IID) client data. To overcome this challenge, we propose a personalized federated learning framework, in which a Takagi–Sugeno (T–S) fuzzy fusion rule is designed. Then, the personalized model is constructed through a structured procedure: fuzzification of model parameter distances, definition of fuzzy rules, fuzzy inference, and defuzzification. Moreover, layer-wise fusion is employed to enhance the precision of aggregation. Evaluations on the Tennessee Eastman (TE) process demonstrate that our method achieves superior fault identification accuracy. The results validate the efficacy of the proposed Fuzzy Rule-Based Federated Layer-wise Fusion (FedFZ) framework in industrial fault diagnosis under heterogeneous data distributions.

## 1. Introduction

Chemical accidents in production facilities can lead to catastrophic incidents, making rapid fault diagnosis and equipment reliability assurance a critical priority. This is particularly relevant for key components such as reactors, condensers, and valve sensors, which operate continuously under extreme temperature and pressure conditions. These components demonstrate pronounced vulnerability to various malfunctions, including reduced feedwater temperature in condensers, abnormal agitator torque, and sticking valves. Consequently, accurate fault diagnosis has become indispensable for ensuring chemical process safety [1].

To address the issue of deep coupling across various stages of chemical production processes, deep learning-based diagnostic methods have been widely adopted in chemical production systems [2]. However, these methods typically rely on the availability of sufficient and uniformly distributed failure data from each production facility [3]. In particular, data collected from different clients frequently exhibit non-independent and identically distributed (non-IID) characteristics [4]. From a measurement science perspective, such non-IID characteristics arise from heterogeneous process measurement conditions, sensor configurations, and operating environments across distributed production units. Additionally, fault data represent sensitive corporate assets that cannot be freely shared. Under this case, the federated learning paradigm—which involves uploading only model features while avoiding direct data exchange—has emerged as a crucial solution [5]. Furthermore, the non-IID challenge has spurred interest in personalized federated learning (PFL) for fault diagnosis scenarios in chemical plants [6,7]. Therefore, this study aims to develop a federated fault diagnosis method that protects industrial data privacy while reducing the negative effect of non-IID client distributions.

However, existing PFL methods including DITTO [8] and PFL-DA [9] still depend on global model sharing, which can degrade performance under non-IID conditions [10]. Although FedAMP [11] addresses this limitation by designing client-specific models, it suffers from two fundamental drawbacks: first, it lacks precise fusion criteria, making performance highly sensitive to hyperparameter selection; second, applying uniform personalization weights across network layers fails to account for their distinct functionalities, thereby compromising feature extraction capability and ultimately limiting diagnostic accuracy [12]. These limitations indicate that chemical process fault diagnosis requires a flexible aggregation mechanism that can handle uncertain client similarity and layer-wise model differences.

To overcome these limitations, we propose a layer-wise personalized federated learning framework incorporating fuzzy fusion rules (FedFZ). Our method treats individual network layers as fusion units [13,14]. The inter-client layer similarity is computed using parameter norm distances, which are subsequently transformed into fusion weights through Takagi–Sugeno (T-S) fuzzy inference. This enables the construction of personalized models for each client, and then guides the optimization of the local model.

To clarify the technical innovation of FedFZ for Tennessee Eastman (TE) process fault diagnosis, the contributions of this paper are reorganized as follows:

- **T–S fuzzy fusion weight generation:** A Takagi–Sugeno fuzzy inference mechanism is designed to map layer-wise inter-client parameter distances into adaptive fusion weights. This provides a smooth and interpretable aggregation rule under non-IID data distributions.
- **Layer-wise personalized aggregation:** Each network layer is treated as an independent fusion unit, and the fusion weights are calculated separately for different layers. This avoids using a uniform aggregation rule for the whole network and improves personalized fusion precision.
- **Client-specific fused model construction:** FedFZ constructs a personalized fused model for each client instead of a single shared global model. This reduces the influence of heterogeneous client updates and improves adaptability in TE process fault diagnosis.

The remainder of this paper is organized as follows. Section 2 reviews fault diagnosis methods in chemical processes and personalized federated learning methods. Section 3 formulates the federated fault diagnosis problem under heterogeneous client data. Section 4 presents the proposed FedFZ framework, including layer-wise fusion, fuzzy rule inference, server-side aggregation, and fuzzy fusion analysis. Section 5 reports the experimental settings, comparative results, ablation studies, and feature visualization on the TE process dataset. Section 6 concludes this paper and discusses future research directions.

## 2. Related Work

### 2.1. Fault Diagnosis Methods in Chemical Processes

Intelligent fault diagnosis has become indispensable for chemical process safety, serving as a critical tool to prevent equipment malfunction escalation and systemic accidents [15,16].

Current fault diagnosis approaches fall into two main categories: statistical analysis and deep learning techniques [17]. Specially, deep learning has generated substantial research interest due to its exceptional feature extraction capabilities [18]. Representative works include Wang’s hybrid framework that integrates LSTM and CNN architectures [19], Han et al.’s orthogonal convolutional autoencoder (OSCAE) enhanced with self-attention mechanisms [20], and Cao et al.’s improved RNN with dual-classifier structure for capturing dynamic temporal features [21]. These methodologies have demonstrated notable diagnostic performance on the TE benchmark process [22].

Recent studies have further explored intelligent fault diagnosis under complex industrial conditions. Li et al. [23] proposed a joint collaborative adaptation network, which combines domain adaptation, adversarial learning, class-aware modeling, and decision fusion for imbalanced and variable-condition bearing diagnosis. Wang et al. [24] used spatiotemporal feature fusion and domain adaptation to reduce distribution differences across working conditions. Lei et al. [25] introduced adversarial open-set domain adaptation to distinguish known and unknown fault samples, while Luo et al. [26] designed an unsupervised domain adaptation network for imbalanced transfer diagnosis. These methods improve diagnosis under complex conditions, but they are mainly developed in centralized or domain adaptation settings.

In contrast, this paper focuses on federated chemical process fault diagnosis, where local data remain private and client collaboration is achieved through layer-wise fuzzy fusion. Existing deep learning diagnosis models still typically assume consistent data distributions [27]. In practical chemical production systems with diverse operational conditions, measured data often exhibit non-IID characteristics [28]. This distribution mismatch hinders the direct application of existing models [29].

### 2.2. Federated Learning Methods

Federated learning (FL) was first introduced through the Federated Averaging (FedAvg) [5] algorithm, which aggregates local model updates on the server side to obtain a global model without sharing raw data. However, its performance degrades significantly under non-IID settings, motivating the development of PFL methods.

To address the non-IID issue of data in heterogeneous environments, personalized federated learning methods have garnered significant attention [30]. Existing studies have demonstrated its significant practical value in industrial fault diagnosis. For instance, HOuaFL mitigates label heterogeneity and operating-condition diversity by introducing data-distillation-based global model initialization and similarity-driven local update selection [31]. This approach enhances diagnostic accuracy while maintaining data privacy. However, HOuaFL lacks clear aggregation logic, making it difficult to clarify how parameter differences across clients at each network layer affect the aggregation process.

The federated learning field has established a systematic research framework supporting industrial fault diagnosis. In model architecture separation, FedRep [32] partitions networks into shared feature extractors and client-specific classifiers. This achieves effective balance between global knowledge transfer and local specialization. PFL-DA [9] implements dual-aggregation mechanisms for shared and personalized layers, significantly improving cross-domain adaptability.

In model aggregation and weight optimization, multiple approaches have emerged. FedProx [29] incorporates proximal terms into local objectives to counter model drift. Ditto [8] jointly optimizes global and local objectives for improved collaboration-specialization balance. FedAMP [11] dynamically adjusts aggregation weights through inter-client similarity measurement. FedFomo [33] enables clients to evaluate model combinations for optimal data distribution matching locally. FedSMU [34] introduces symbolized updates with momentum compensation, effectively reducing aggregation variance under heterogeneous conditions.

In recent years, adaptive and ensemble approaches have also emerged. FedALA [35] proposes an adaptive local aggregation strategy based on data characteristics. pFedLA [13] enables end-to-end optimization of trainable local aggregation weights for finer-grained personalization. FedDBE [36] enhances global stability by integrating multiple rounds of local model snapshots. FedBABU [37] updates only feature extractors during federation while fine-tuning classifiers locally, improving both global representation and personalization.

Despite progress in mitigating data heterogeneity and improving diagnostic accuracy, existing methods remain limited for chemical processes with complex dynamics. Most approaches rely on single global similarity measures or uniform aggregation rules. This restricts their ability to capture fine-grained semantic differences across network layers [38]. The aggregation logic often remains unclear, hindering understanding of how layer-wise parameter differences affect federation outcomes. To address this, we propose a fuzzy rule-based hierarchical fusion strategy that automatically computes layer-wise aggregation weights from inter-client parameter distances [39], improving fault diagnosis under strongly non-IID conditions.

## 3. Problem Formulation

Formally, assume that the *n*-th client maintains a neural network model with parameters

(1) $$v_{n}:={\left[v_{n,0},\cdots ,v_{n,l},\cdots ,v_{n,L}\right]}^{T},$$

where $v_{n,l}$ denotes the parameters of the *l*-th layer, and *L* is the total number of layers. The global optimization objective of FL can be written as

(2) $$\min G\left(V\right):=\sum_{n=1}^{N}F_{n}\left(v_{n}\right),$$

where $V=[v_{1},\ldots ,v_{N}]$ represents the collection of client models, and $F_{n}\left(v_{n}\right)$ is the local loss function computed on client *n*’s dataset $D_{n}$.

In chemical production processes, the data collected from distributed units often exhibit significant heterogeneity arising from variations in operation modes, process couplings, and measurement conditions. Such heterogeneity causes locally trained model parameters to deviate in both magnitude and distribution, making direct global aggregation suboptimal. Moreover, different layers of neural networks capture features of varying abstraction levels, leading to inconsistent sensitivity to non-IID conditions across layers.

To address these challenges, this paper proposes FedFZ, a fuzzy rule-based hierarchical fusion framework that integrates inter-client parameter distances into a T–S fuzzy inference system. FedFZ adaptively determines layer-wise aggregation weights, effectively mitigating heterogeneity and similarity uncertainty in non-IID industrial scenarios.

## 4. Multi-Layer Fusion Method Based on Fuzzy Rules

**Figure 1:**
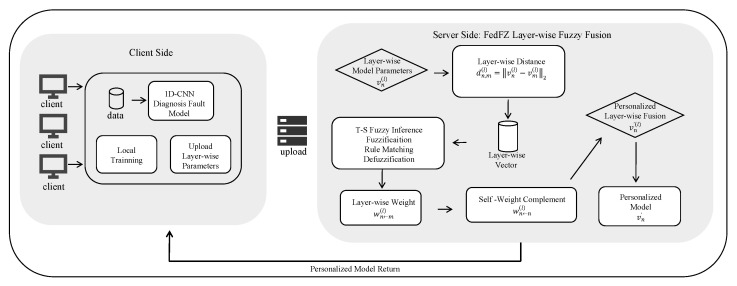
Framework of the proposed FedFZ method.

This section introduces the FedFZ framework for fault diagnosis in chemical production and manufacturing equipment, as shown in Figure 1. Each client represents a production unit and builds a CNN fault diagnosis model using its own process data (e.g., temperature, pressure, and flow signals) before uploading the model to the server. During the fusion phase, the server treats network layers as fusion units and constructs a fuzzy fusion system based on the parameter distance of each client’s corresponding layer, thereby obtaining personalized fault diagnosis models for each client. Different network layer types may exhibit distinct sensitivities to non-IID data. Motivated by this observation, the proposed framework adopts a layer-wise fusion strategy to accommodate the heterogeneous roles of convolution and fully connected layers. This design follows the idea that unified fusion is suitable for the feature extraction layer, while personalized fusion is appropriate for the task-specific layer [40].

Finally, the server pushes the model to the corresponding client for fault diagnosis. Unlike conventional personalization methods that rely on regularization, FedFZ introduces a fuzzy rule-driven hierarchical fusion mechanism, which adaptively assigns fusion weights based on inter-client divergence. This design mitigates uncertainties in the inter-model distance and enhances the accuracy of fault diagnosis in complex industrial production scenarios.

### 4.1. Client-Side Training

We have found that, under conditions of sufficient data, convolutional neural networks are capable of meeting the accuracy requirements for equipment fault diagnosis in manufacturing production. Accordingly, in the proposed FedFZ framework, each client employs a CNN-based fault diagnosis model as its local learner, which is trained on private industrial data for subsequent layer-wise fuzzy fusion. Each client updates its local model by minimizing the standard cross-entropy loss:

(3) $$L_{n}\left(v_{n}\right)=\frac{1}{|D_{n}|}\sum_{\left(x_{i},y_{i}\right)\in D_{n}}\ell \left(f\left(v_{n},x_{i}\right),y_{i}\right),$$

where $D_{n}$ denotes the local dataset of client *n*, $|D_{n}|$ is the number of samples, $f(v_{n},x_{i})$ represents the model prediction for input $x_{i}$, $y_{i}$ is the corresponding fault label, and ℓ(·) is the cross-entropy loss function.

### 4.2. Layer-Wise Fusion Strategy

Although the method of dividing network functions can improve the accuracy of fault diagnosis [37], it fails to account for differences across network layers. To further improve model fusion precision, we enforce a federated learning framework that uses layers as fusion units. For client *n*, its layer-wise aggregation weights are defined as:

(4) $$W_{n}=\left[w_{n,1},w_{n,2},\ldots ,w_{n,L}\right],$$

where *L* is the total number of layers, and $w_{n,l}$ denotes the weight assigned to the *l*-th layer of client *n*. The fused model parameters are expressed as:

(5) $$v_{n}^{\prime }=\mathrm{Diag}\left(V\cdot W_{n}\right),$$

where *V* is the matrix of all clients’ layer-wise parameters, and Diag(·) denotes element-wise aggregation across layers.

### 4.3. Fuzzy Inference-Based Fusion Rules

To facilitate the subsequent layer-wise aggregation, we define a fuzzy inference operator, denoted as FuzzyRule(·), which adaptively maps the inter-client parameter distance to a fusion weight. This operator represents the core of the proposed hierarchical fusion framework, where each pair of client parameters is processed through a fuzzy reasoning mechanism to determine their mutual influence. Formally, for the *l*-th layer of clients *k* and *j*, the operator outputs:

(6) $$w_{k\leftarrow j}^{\left(l\right)}=\mathrm{FuzzyRule}\;\left(v_{k}^{\left(l\right)},\;v_{j}^{\left(l\right)}\right),$$

where $w_{k\leftarrow j}^{\left(l\right)}$ denotes the contribution weight of client *j* to client *k* at layer *l*. The internal construction of FuzzyRule(·) is introduced as follows.

Due to uncertainties in parameter metric calculations between models, the fused diagnostic model exhibits bias. Inspired by fuzzy logic, this paper proposes a fusion method based on fuzzy inference [41]. The procedure comprises four steps: fuzzification, rule definition, fuzzy inference, and defuzzification, as described below.

#### 4.3.1. Fuzzification

For each layer *l*, the inter-client distance $d_{k,j}^{\left(l\right)}$ between clients *k* and *j* is computed using the Euclidean metric:

(7) $$d_{k,j}^{\left(l\right)}=\exp\;\left(-\;\frac{{∥v_{k}^{\left(l\right)}-v_{j}^{\left(l\right)}∥}_{2}^{2}}{2\sigma_{d}^{2}}\right),$$

where $v_{k}^{\left(l\right)}$ and $v_{j}^{\left(l\right)}$ denote the parameter vectors of clients *k* and *j* at layer *l*, respectively, and $\sigma_{d}$ controls the decay rate of the distance. The computed value is denoted as $x:=d_{k,j}^{\left(l\right)}$ and serves as the input to the Gaussian membership functions,

(8) $$\mu_{i}\left(x\right)=\exp\;\left[-\frac{{\left(x-c_{i}\right)}^{2}}{2\sigma_{\mu ,i}^{2}}\right],\;i=1,\ldots ,S,$$

where $c_{i}$ and $\sigma_{\mu ,i}$ denote the center and spread of the *i*-th fuzzy set, respectively. The membership degrees ${\left\{\mu_{i}\left(x\right)\right\}}_{i=1}^{S}$ provide the quantitative basis for the subsequent fuzzy rule and defuzzification, where they will be combined with the representative weights $w_{i}$ of each fuzzy set and normalized to obtain the final layer-wise aggregation weight for each client.

#### 4.3.2. Fuzzy Rule

Based on the T–S fuzzy inference, fuzzy rules are formulated to assign representative weights according to the distance level. For clarity, the following five-rule form is used only as an illustrative example to describe the construction of fuzzy rules. In the main FedFZ implementation, the final number of fuzzy segments is set to S=7, which is selected according to the parameter sensitivity results in Section 5.3.2. Specifically, a fuzzy set with five inference rules is defined as

(9) $$\left\{\begin{array}{cc} \mathrm{Rule}\;1: & \mathrm{IF}\;x\;\mathrm{is}\;A_{1}\left(c_{1},\sigma_{\mu ,1}\right)\;\mathrm{THEN}\;w_{1},\\ \mathrm{Rule}\;2: & \mathrm{IF}\;x\;\mathrm{is}\;A_{2}\left(c_{2},\sigma_{\mu ,2}\right)\;\mathrm{THEN}\;w_{2},\\ \mathrm{Rule}\;3: & \mathrm{IF}\;x\;\mathrm{is}\;A_{3}\left(c_{3},\sigma_{\mu ,3}\right)\;\mathrm{THEN}\;w_{3},\\ \mathrm{Rule}\;4: & \mathrm{IF}\;x\;\mathrm{is}\;A_{4}\left(c_{4},\sigma_{\mu ,4}\right)\;\mathrm{THEN}\;w_{4},\\ \mathrm{Rule}\;5: & \mathrm{IF}\;x\;\mathrm{is}\;A_{5}\left(c_{5},\sigma_{\mu ,5}\right)\;\mathrm{THEN}\;w_{5}. \end{array}\right.$$

Here, $w_{i}$ (i=1,…,5) denotes the predefined representative fusion weight associated with the *i*-th fuzzy rule, which will later be combined with the corresponding membership degree $\mu_{i}\left(x\right)$ during the defuzzification stage. *x* represents the normalized inter-client distance, and $A_{i}$ (i=1,…,5) denotes the Gaussian fuzzy set (e.g., very small, small, medium, large, very large). For each input *x*, the fuzzification step provides the membership degrees $\mu_{i}\left(x\right)$ of these sets, so that every rule contributes a pair $\left(\mu_{i}\left(x\right),w_{i}\right)$ to the aggregation. Together, these rules establish a smooth mapping from inter-client divergence to layer-wise aggregation weights, ensuring that clients with smaller distances exert stronger influence, while those with larger divergences are downweighted.

#### 4.3.3. Fuzzy Weight Computation

Based on the membership degrees $\mu_{i}\left(x\right)$ of all fuzzy sets and their representative weights $w_{i}$ from the preceding stages, the layer-wise aggregation weight is computed by

(10) $$w_{o}=\frac{\sum_{i=1}^{S}\mu_{i}\left(x\right)\;w_{i}}{\sum_{i=1}^{S}\mu_{i}\left(x\right)}.$$

The representative weights $w_{i}$ are treated as rule-level hyperparameters rather than trainable parameters in the local optimization process. Their setting follows the monotonic relationship between inter-client similarity and aggregation contribution. A smaller parameter distance indicates higher client similarity and is assigned a larger fusion contribution, whereas a larger distance indicates stronger client divergence and is assigned a smaller contribution. This design keeps the fuzzy fusion process interpretable and avoids introducing extra optimization variables into local training. The influence of these manually predefined weights is further examined through the sensitivity analysis in Section 5.3.2. This operation simultaneously integrates the influence of all fuzzy rules and produces a crisp aggregation weight $w_{o}$ for each layer. As a result, clients with smaller distances are assigned higher $w_{o}$, whereas those with larger divergences are suppressed, thereby alleviating the adverse effect of client update heterogeneity on the federated aggregation process.

### 4.4. Server-Side Fusion and Aggregation

For each layer *l*, the server first computes, for every client *n*, the fuzzy fusion weights with respect to all other clients m≠n:

(11) $$w_{n\leftarrow m}^{\left(l\right)}=\mathrm{FuzzyRule}\left(v_{n}^{\left(l\right)},\;v_{m}^{\left(l\right)}\right),\;m\neq n.$$

Here, $w_{n\leftarrow m}^{\left(l\right)}$ denotes the contribution weight from source client *m* to target client *n* at layer *l*. The self-weight for client *n* at layer *l* is derived by complementarity:

(12) $$w_{n\leftarrow n}^{\left(l\right)}=1-\sum_{m\neq n}w_{n\leftarrow m}^{\left(l\right)}.$$

The self-weight represents the remaining contribution assigned to client *n* itself. Therefore, the contributions from other clients and the self-weight are normalized to one for the personalized aggregation of client *n* at layer *l*. Since the fuzzy fusion weights are non-negative and bounded in the fusion process, $w_{n\leftarrow n}^{\left(l\right)}$ also remains non-negative. Finally, the personalized aggregated parameter of layer *l* for client *n* is computed as

(13) $$v_{n}^{\prime (l)}=\sum_{m=1}^{N}w_{n\leftarrow m}^{\left(l\right)}\cdot v_{m}^{\left(l\right)}.$$

Then, collecting all layer-wise outputs ${\left\{v_{n}^{\prime (l)}\right\}}_{l=1}^{L}$ yields the complete fused model, which is computed using the same formulation as defined in Equation (5).

The hierarchical fuzzy fusion adaptively integrates similarity-weighted contributions from all clients, ensuring robust and personalized model aggregation even under non-IID client data distributions.

### 4.5. Analysis of Fuzzy Fusion

The fuzzy fusion strategy in FedFZ aims to provide a smooth and robust mapping from inter-client parameter distances to aggregation weights under non-IID conditions. In practical federated learning, parameter distances are often noisy and non-stationary due to stochastic local updates and heterogeneous data distributions, making direct distance-based weighting or hard thresholding prone to unstable aggregation.

**Figure 2:**
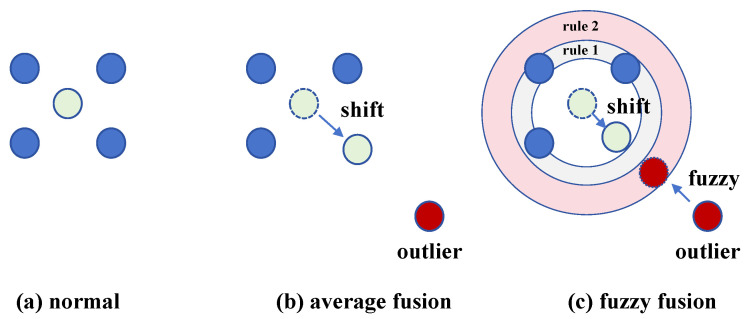
Illustration of the TS fuzzy-rule-based fusion mechanism under abnormal client updates.

The working mechanism of the proposed fuzzy fusion is illustrated in Figure 2. As shown in Figure 2a, direct averaging can be significantly biased by abnormal client updates, leading to a noticeable shift of the aggregation center. Instead of explicitly detecting or discarding outliers, the proposed method softly partitions the distance space into fuzzy regions, as shown in Figure 2b, and continuously adjusts aggregation weights through TS fuzzy inference [42]. This results in a smooth distance-to-weight mapping Figure 2c, where abnormal updates are assigned reduced influence, while the relative similarity ordering among clients are preserved. The results demonstrate that federated learning with fuzzy inference can enhance model robustness while maintaining relative improvements.

The representative weights $w_{i}$ and the membership function parameters are manually predefined according to the design objective of fuzzy fusion. The weights $w_{i}$ are treated as rule-level hyperparameters rather than optimizable parameters, which is consistent with common design practices in interpretable fuzzy rule-based systems [43]. Their selection follows the monotonic correspondence between client similarity and aggregation contribution, while maintaining a moderate dynamic range to avoid unstable aggregation. The membership centers and widths regulate the smoothness and sensitivity of the distance-to-weight mapping. From a theoretical perspective, the proposed fuzzy fusion follows the classical Takagi–Sugeno fuzzy inference paradigm for uncertainty handling in complex systems [44,45]. Therefore, these parameters are not learned by a data-driven optimization algorithm, but are selected based on interpretable fuzzy rule design and empirically verified through the sensitivity analysis in Section 5.3.2.

FedFZ is related to FedAMP [11], since both methods follow the principle of similarity-based personalized collaboration. FedAMP encourages stronger collaboration among clients with higher model similarity, while FedFZ extends this idea from model-level aggregation to layer-wise fuzzy aggregation. For each client and each layer, the fuzzy fusion weights are non-negative, and the complementary self-weight makes the aggregation weights form a normalized weighted combination of client parameters. This structure prevents parameter amplification caused by unbounded aggregation weights and provides a basic stability condition for the fusion process. Since the 1D-CNN used in this study leads to a non-convex optimization problem, this paper does not claim a strict closed-form convergence rate. Instead, the convergence behavior of FedFZ is evaluated empirically under the Dirichlet non-IID setting with α=0.1. The accuracy curves, confidence intervals, and ablation results show that FedFZ achieves stable convergence and maintains better diagnostic performance than the comparison methods under heterogeneous client data. The overall procedure of the proposed FedFZ method is summarized in Algorithm 1.
**Algorithm 1** FedFZ: Fuzzy Rule-Based Federated Layer-wise Fusion  1:**Initialize:** number of clients *N*, datasets $D_{k}$, layers *L*, rounds *R*.  2:**for** t=1 **to** *R* **do**  3:    **Server (no global model stored):**  4:    Collect ${\left\{v_{k}^{\left(l\right)}\right\}}_{k=1}^{N}$ from all clients.  5:    **for** each client *n* **do**  6:        **for** l=1 **to** *L* **do**  7:           Compute $w_{n\leftarrow m}^{\left(l\right)}$ for all m≠n.  8:           Set $w_{n\leftarrow n}^{\left(l\right)}\leftarrow 1-\sum_{m\neq n}w_{n\leftarrow m}^{\left(l\right)}$.  9:           $v_{n}^{\prime (l)}\leftarrow \sum_{m=1}^{N}w_{n\leftarrow m}^{\left(l\right)}\cdot v_{m}^{\left(l\right)}$10:        **end for**11:        Assemble $v_{n}^{\prime }\leftarrow {\left\{v_{n}^{\prime (l)}\right\}}_{l=1}^{L}$ and send to client *n*.12:    **end for**13:    **Clients (parallel):** receive $v_{n}^{\prime }$, fine-tune on $D_{n}$, upload ${\left\{v_{n}^{\left(l\right)}\right\}}_{l=1}^{L}$.14:**end for**15:**Output:** personalized models $v_{n}^{\prime }$ for all clients.

## 5. Experimental Results and Analysis

### 5.1. Datasets and Experimental Settings

To verify the effectiveness of the proposed FedFZ framework, this paper conducts experiments using the Tennessee Eastman (TE) process dataset, a widely adopted benchmark in process fault diagnosis.

**Figure 3:**
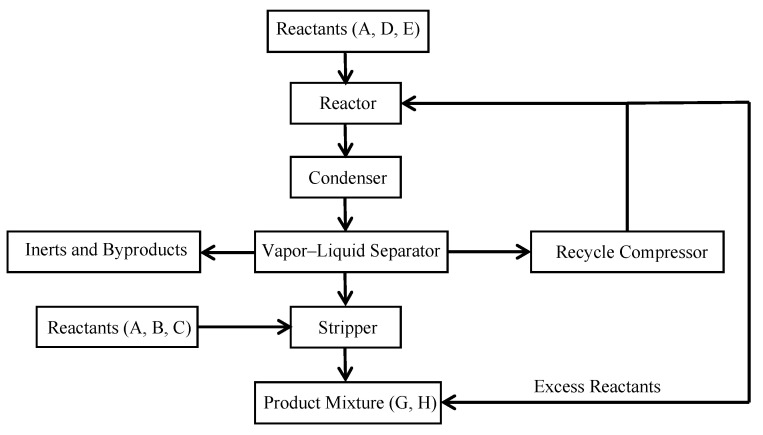
Process flow diagram of the Tennessee Eastman (TE) system.

The TE process simulates a typical chemical production system consisting of multiple interconnected units, including a reactor, condenser, vapor-liquid separator, and recycle compressor, as illustrated in Figure 3. These units exhibit strong coupling and complex feedback loops, making the fault diagnosis task particularly challenging and representative of real-world industrial environments. The TE process was selected as the main validation dataset because it provides a representative and reproducible benchmark for chemical process fault diagnosis. Although the TE process is a simulated benchmark, it retains several key characteristics of chemical production systems, including multivariable measurements, strong process coupling, feedback control loops, and diverse fault patterns. These characteristics make it suitable for evaluating federated fault diagnosis methods under heterogeneous industrial data distributions.

This paper selects nine representative fault types from the standard TE fault library. These are combined with the normal operating condition to construct a 10-class classification task. These fault types encompass diverse failure modes, including actuator malfunctions, temperature anomalies, and sensor biases, and cover typical patterns such as step changes, random variations, and bias faults, as summarized in Table 1.

The original dataset comprises a wide array of sensor measurements and control variables. To reduce dimensionality and alleviate model complexity, a LightGBM-based feature selection procedure is adopted. Specifically, a LightGBM classifier [46] is trained to rank features according to their importance, and a 5-fold stratified cross-validation is conducted with progressively larger top-ranked feature subsets. The subset size yielding the best validation accuracy is selected, resulting in 33 retained input features for subsequent model training. After feature selection, each sampling time point is represented by a 33-dimensional feature vector.

The resulting time-series data are further segmented using a temporal sliding window approach. In this study, the window length is set to 200 time steps, and the stride is set to 25 time steps. Therefore, each windowed sample has an input size of 33×200. The label of each window is assigned according to the operating condition or fault type of the corresponding TE sequence. Each windowed segment is treated as an independent training or inference sample.

The federated learning task is designed as a cross-silo fault diagnosis scenario, where each client represents an independent chemical production unit and keeps its local data private. To simulate the data heterogeneity commonly encountered in federated learning environments, the windowed samples are partitioned among 10 clients using a Dirichlet distribution with concentration parameter α=0.1, thus creating a highly non-IID data distribution across clients. In each communication round, clients train local 1D-CNNs using their own windowed samples and upload only model parameters. The server performs layer-wise fuzzy fusion and returns personalized models to clients. Final accuracy in the main comparison is reported with the corresponding 95% confidence interval over repeated runs. Precision, Recall, and F1-score are also reported to further evaluate the overall diagnostic performance.

The main training hyperparameters are further specified to improve experimental reproducibility. In the federated experiments, the total number of communication rounds is set to 100. Each client trains the local 1D-CNN model using stochastic gradient descent (SGD), with a learning rate of 0.05 and a local batch size of 16. The cross-entropy loss is used as the classification objective. All comparison methods use the same CNN architecture, optimizer, learning rate, batch size, number of clients, and communication rounds to ensure a fair comparison.

All experiments are conducted on a high-performance server equipped with an NVIDIA RTX 3090 GPU, 512 GB of RAM, and running Ubuntu 20.04.3 LTS. The entire training and evaluation pipeline is implemented using PyTorch 1.9.

### 5.2. Performance Comparison on the TE Process Dataset

To evaluate the effectiveness of the proposed FedFZ method in fault diagnosis for the TE process, we conduct comparative experiments with several personalized federated learning algorithms, including FedAvg [5], FedAMP [11], Ditto [8], PFL-DA [9], FedDBE [36], FedALA [35], and FedSMU [34]. The adopted model architecture is a one-dimensional convolutional neural network (CNN) consisting of two convolutional layers followed by two fully connected layers. The detailed architecture of the adopted CNN is summarized in Table 2. The input dimension is set to 33 channels with a sequence length of 200, and the number of clients is fixed at N=10. After federated training, the performance is evaluated on the test sets of all clients.

In addition to classification accuracy, we further report Precision, Recall, and F1-score to provide a more comprehensive evaluation of diagnostic performance under heterogeneous data distributions.

**Figure 4:**
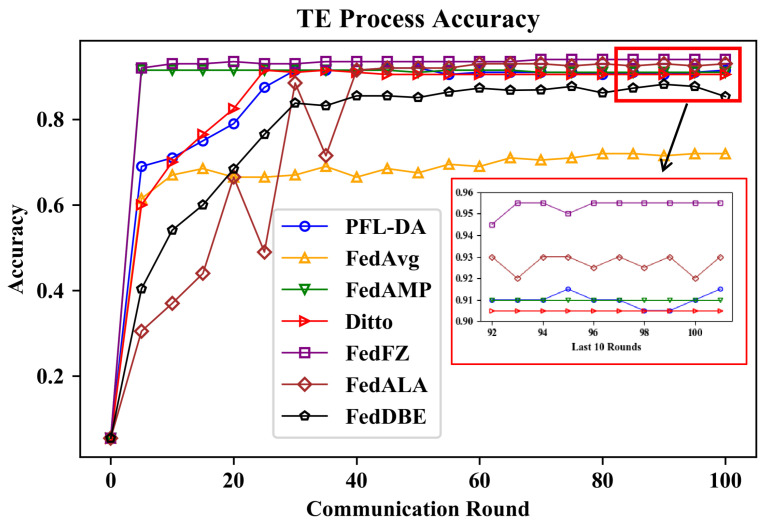
Accuracy comparison on the TE dataset.

The comparative results in Figure 4 show the evolution of test accuracy during training. FedFZ exceeds 90% accuracy in the early communication rounds and reaches the highest accuracy of 95.5% at convergence. FedALA obtains the second-best accuracy of 93.5%. PFL-DA and FedAMP both reach 91.5%, followed by Ditto with 90.5%. FedDBE, FedSMU, and FedAvg show lower final accuracies. These results indicate that FedFZ achieves better diagnostic performance under the non-IID TE data partition.

In contrast, FedFZ maintains a high and stable level of accuracy throughout the training process. Compared with FedAMP, whose performance degrades under highly non-IID conditions due to instability in client similarity modeling, FedFZ effectively mitigates such fluctuations by introducing fuzzy rule-based hierarchical fusion. This mechanism adaptively assigns aggregation weights according to inter-client distance, smoothing the fusion process and alleviating error accumulation during training.

In terms of communication efficiency, FedFZ achieves comparable or better accuracy within fewer communication rounds, demonstrating faster convergence with reduced communication overhead. This advantage is particularly valuable in industrial scenarios where communication resources are limited and client heterogeneity is significant. Overall, FedFZ demonstrates superior test accuracy and communication efficiency, confirming its effectiveness and potential applicability in handling Non-IID industrial data scenarios.

The quantitative comparison results are further summarized in Table 3.

Consistent trends are observed across Accuracy, Precision, Recall, and F1-score, indicating that the performance gains of FedFZ are not limited to a single metric but extend to overall classification quality. The added confidence intervals further support the stability of the reported accuracy improvement.

### 5.3. Ablation Study Results

To further verify the effectiveness of the proposed framework, we conduct additional experiments from several aspects, including comparison with centralized training, parameter sensitivity, distance metric robustness, component contribution, and layer-type-specific fusion. These experiments clarify the performance gap between centralized and federated training, and further analyze the role of each design under the non-IID TE data partition.

#### 5.3.1. Comparison with Centralized Training

To quantify the performance gap between federated learning and fully centralized training, we add a Centralized-CNN baseline. This baseline uses the same CNN architecture as FedFZ, while all TE training samples are collected together for model training. Therefore, Centralized-CNN can access the complete data distribution and is used as an ideal upper-bound reference. FedAvg and FedFZ are trained under the federated setting, where raw data remain on local clients and only model parameters are exchanged.

As shown in Table 4, Centralized-CNN achieves the highest accuracy of 98.5%, because it directly uses all training samples. FedFZ reaches 95.5% under the federated setting, with a gap of 3.0 percentage points compared with Centralized-CNN. This result shows that FedFZ still maintains competitive diagnostic performance without direct raw data sharing. Compared with FedAvg, FedFZ further reduces the performance loss caused by non-IID data through fuzzy rule-based layer-wise fusion.

#### 5.3.2. Parameter Sensitivity and Distance Robustness

In federated learning under non-IID conditions, inter-client parameter distances do not distribute uniformly. As training proceeds, repeated aggregation pulls most client models toward a common region, forming a concentrated intermediate distance range, while a small number of clients remain significantly deviated due to data imbalance or distribution shifts, resulting in a long-tailed distance distribution.

To improve reproducibility, the main fuzzy hyperparameters used in the final FedFZ implementation are summarized in Table 5. These settings include the Gaussian membership centers and spreads, the representative weights, and the distance scaling parameter, and are manually predefined based on the monotonic relationship between inter-client similarity and aggregation contribution.

**Figure 5:**
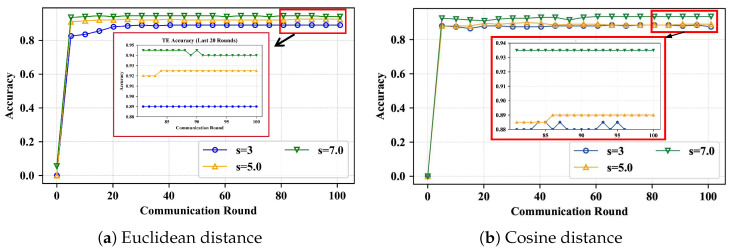
Comparison of accuracy curves under Euclidean and Cosine distances on the TE dataset.

Figure 5a,b show the convergence curves for different values of *S* under Euclidean and cosine distances, respectively. Increasing *S* consistently improves both accuracy and convergence stability. In particular, the seven-segment rule achieves the best performance, yielding final accuracies of 94.5% with Euclidean distance and 93.5% with cosine distance, which are significantly higher than those of the three-segment and five-segment rules. These results demonstrate that finer segmentation better captures inter-client differences and thereby enhances aggregation effectiveness. In these experiments, the representative weights $w_{i}$ for the three-segment, five-segment, and seven-segment rules are pre-assigned and empirically tuned rather than learned automatically; their specific values are three segments [0.001, 0.01, 0.1], five segments [0.001, 0.005, 0.0025, 0.05, 0.1], and seven segments [0.001, 0.008, 0.01, 0.027, 0.03, 0.06, 0.1], respectively. These results demonstrate that seven fuzzy rule segments achieve the best performance under the considered non-IID setting, reflecting a practical balance between representation granularity and robustness, which is consistent with common design principles in fuzzy rule-based systems [43].

**Figure 6:**
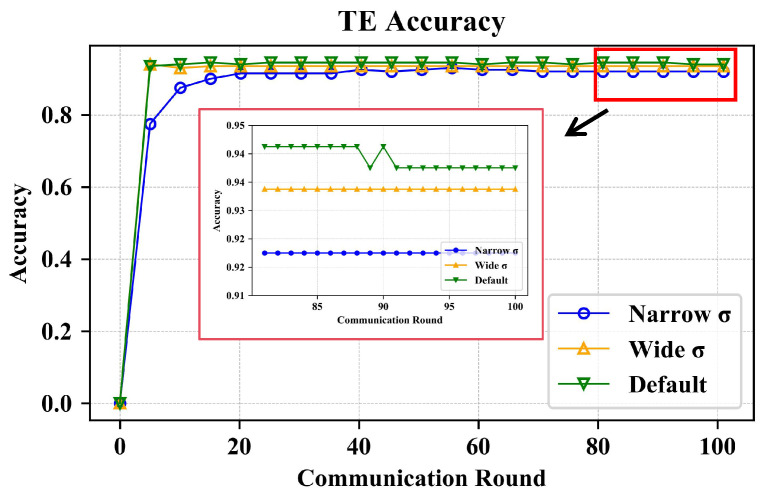
Accuracy comparison under different membership function widths.

Furthermore, Figure 6 and Table 6 present the results under different membership function configurations. Both the center shift (*c*) and the sigma scale (σ) affect model performance. Here σ denotes a scaling factor related to the default width, rather than an absolute variance. A smaller scaling factor (e.g., 0.5) effectively narrows the membership functions, accelerating early convergence but potentially reducing final accuracy, whereas a larger scaling factor (e.g., 1.5) widens the functions, improving stability at the cost of slower convergence. Small shifts of *c* lead to accuracy fluctuations, and when combined with width scaling the performance degrades further. Overall, the default configuration F1 (c=0,σ=1.0) achieves the highest accuracy (94.5%) with stable convergence, confirming the soundness of our parameter choice. These results indicate that, under the considered experimental settings, the default membership configuration achieves the highest final accuracy with stable convergence, while moderate variations of the membership parameters lead to observable but limited performance degradation.

These sensitivity results provide empirical support for the adopted fuzzy parameter setting. The seven-segment rule improves the description of inter-client distance differences, while the default membership configuration with c=0 and σ=1.0 provides a stable mapping without excessive sensitivity. Therefore, the final setting is selected according to both fuzzy rule design principles and empirical validation, rather than automatic parameter learning.

It is also worth noting that the superior performance of the seven-segment fuzzy rule does not imply that further increasing the number of segments, such as S=9 or S=10, would continuously improve performance. Increasing the segmentation granularity narrows the effective membership regions, which is conceptually similar to reducing σ. As shown in the ablation study, overly narrow membership functions make the fusion process more sensitive to minor inter-client differences and may degrade performance. Therefore, S=7 achieves a favorable balance between discriminability and stability, while further segmentation may introduce unnecessary fluctuations without additional performance gain.

#### 5.3.3. Contribution of Fuzzy Inference and Layered Fusion

To further clarify the respective contributions of fuzzy inference and layered fusion, we conduct additional ablation experiments by selectively removing these two components while keeping all other settings unchanged.

Global-Fuzzy removes layer-wise fusion, while Layered-Avg removes fuzzy inference. As shown in Table 7, removing either fuzzy inference or layered fusion leads to clear performance degradation, indicating that the performance gain of FedFZ arises from the joint contribution of both components.

#### 5.3.4. Effect of Layer-Type-Specific Fusion Strategy

We examine whether different network layer types exhibit distinct fusion requirements under non-IID conditions by comparing uniform layer-wise fusion with a layer-type-specific fusion strategy.

The corresponding ablation results are summarized in Table 8.

These results provide empirical evidence that convolutional layers may prefer uniform aggregation, whereas fully connected layers may benefit from stronger personalization under non-IID conditions.

### 5.4. Feature Visualization Analysis

**Figure 7:**
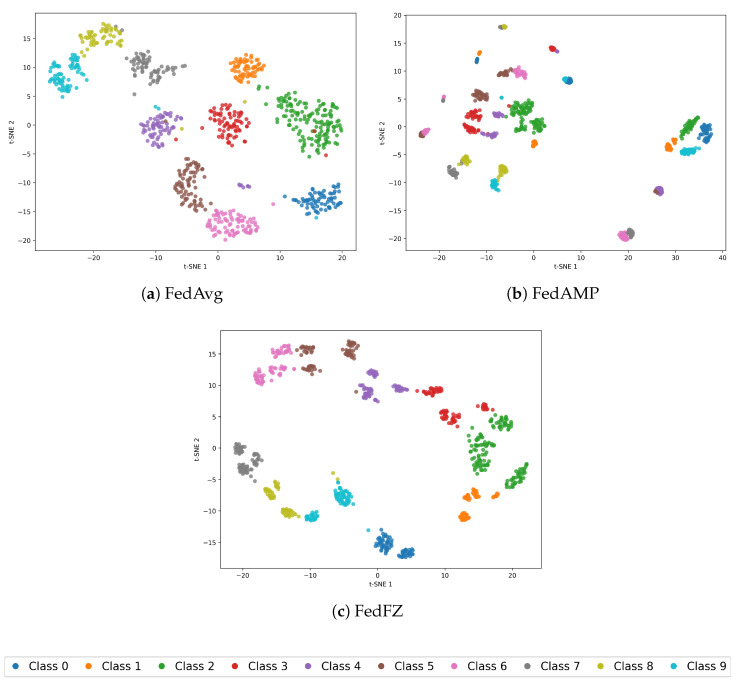
Comparison of t-SNE visualizations under different methods.

To further evaluate the feature representation capability of different methods, we conduct t-SNE visualization on the client-side test features after model convergence, as shown in Figure 7.

The results show that FedAvg produces highly overlapped distributions across classes, with indistinct boundaries and severe inter-class mixing, indicating weak class separability. FedAMP improves clustering through personalized aggregation, and some categories achieve partial separation, though overlaps and dispersion remain.

In contrast, the proposed FedFZ yields more compact and clearly separated clusters in the t-SNE space. Most classes form well-defined groupings, demonstrating that FedFZ enables clients to learn discriminative features with improved consistency across heterogeneous data. Such compact and well-separated clusters are particularly valuable for fault isolation in industrial applications.

### 5.5. Discussion on Dataset Representativeness and Generalization

Although the experiments are conducted on the TE process dataset, the proposed FedFZ framework is not limited to this specific process. The core mechanism of FedFZ depends on layer-wise parameter distances and fuzzy rule-based aggregation weights, rather than process-specific physical assumptions. Therefore, the proposed method has potential applicability to other distributed industrial fault diagnosis tasks with non-IID data, including equipment monitoring, process anomaly diagnosis, and multivariable sensor-based fault classification.

At the same time, the current validation is still limited to one benchmark process dataset. Real industrial datasets may involve stronger noise, missing values, changing operating modes, and more complex fault evolution patterns. These factors may affect the stability of inter-client distance measurement and fuzzy aggregation. Therefore, the current results should be interpreted as evidence obtained from a representative benchmark rather than as a complete validation across all industrial processes. The main purpose of this study is to verify whether fuzzy rule-based layer-wise fusion can improve personalized federated fault diagnosis under a controlled and reproducible non-IID setting. Future work will extend the evaluation to real plant data and additional public industrial process datasets to further examine the applicability of FedFZ under more diverse operating conditions.

## 6. Conclusions

This paper introduces FedFZ, a fuzzy rule-based hierarchical fusion framework for personalized federated learning, designed to address data heterogeneity in industrial fault diagnosis. The proposed method systematically computes layer-wise aggregation weights, enhancing both feature consistency and task-specific adaptation. Empirical evaluations on the Tennessee Eastman process demonstrate FedFZ’s superiority over existing approaches in convergence speed and classification accuracy. However, the current study still has several limitations. A performance gap remains between FedFZ and centralized models, and the present validation is mainly based on the TE benchmark process. Although the TE process is representative and reproducible for chemical process fault diagnosis, a single benchmark cannot fully reflect the diversity of real industrial scenarios, such as different plant structures, operating modes, noise levels, and fault evolution patterns. Future research will extend FedFZ to real industrial datasets and other public process datasets to further evaluate its applicability under more diverse operating conditions.

## Figures and Tables

**Table 1:** Selected 10 categories in the TE process classification task.

Class ID	Fault Description (TE Official Fault IDV)	Fault Type
0	Normal operation	Normal
2	Composition variation in reactant B (IDV 4)	Step
3	Temperature variation of stream B (IDV 2)	Step
4	Temperature variation of reactor cooling water inlet (IDV 5)	Step
5	Temperature variation of condenser cooling water inlet (IDV 6)	Step
8	Abnormal agitator torque (IDV 9)	Random
9	Cooling water supply temperature drop (IDV 10)	Random
10	Valve failure (IDV 11)	Random
11	Valve sticking (IDV 13)	Sticking
13	Agitator position bias (IDV 16)	Bias

*Note:* IDV denotes Independent Disturbance Variable, the standard fault numbering in the TE process. Only nine representative faults and one normal condition are used, and the Class ID values are re-indexed, hence not consecutive.

**Table 2:** Architecture of the CNN used in the experiments.

Layer	Output Size	Description
Input	33×200	Selected input sequence
Conv block 1	32×100	Conv1D + pooling
Conv block 2	64×50	Conv1D + pooling
Fully connected	512	Feature transformation
Output layer	10	Fault classification

**Table 3:** Comparison of multiple evaluation metrics on the TE dataset.

Method	Accuracy (%, 95% CI)	Precision	Recall	F1-Score
FedAvg	76.0 ± 1.24	0.7914	0.7400	0.7643
PFL-DA	91.5 ± 0.82	0.9100	0.9000	0.9050
FedAMP	91.5 ± 0.78	0.9150	0.9100	0.9130
Ditto	90.5 ± 0.86	0.9071	0.9028	0.9050
FedDBE	85.0 ± 1.12	0.8760	0.8450	0.8593
FedALA	93.5 ± 0.64	0.9347	0.9300	0.9323
FedSMU	83.0 ± 1.20	0.8050	0.7800	0.7925
FedFZ	**95.5 ± 0.39**	**0.9462**	**0.9438**	**0.9450**

*Note:* Bold values in the data rows indicate the best performance among all compared methods.

**Table 4:** Comparison with centralized training on the TE dataset.

Method	Training Paradigm	Raw Data Sharing	Accuracy (%)
Centralized-CNN	Centralized	Required	98.5
FedAvg	Federated	Not required	76.1
FedFZ	Federated	Not required	95.5

**Table 5:** Main fuzzy hyperparameters used in FedFZ.

Hyperparameter	Setting
Fuzzy inference type	T–S fuzzy inference
Membership function	Gaussian function
Fuzzy input domain	x∈[0,1]
Distance scaling parameter	$\sigma_{d}=1.0\times 10^{4}$
Tested rule segmentations	S=3,5,7
Final rule segmentation	S=7
Final Gaussian centers $c_{i}$	[0.015,0.055,0.120,0.200,0.380,0.600,0.860]
Final Gaussian spreads $\sigma_{\mu ,i}$	[0.015,0.025,0.040,0.060,0.100,0.120,0.140]
Final representative weights $w_{i}$	[0.001,0.008,0.010,0.027,0.030,0.060,0.100]

**Table 6:** Ablation results of membership function parameters (mean *c* and spread σ adjustment).

Config ID	Mean Shift *c*	Sigma Scale σ	Accuracy (%)	Remarks
F1 (Default)	0.0	1.0	94.5	Default
F2	+0.05	1.0	93.0	Right shift
F3	−0.05	1.0	93.5	Left shift
F4	0.0	1.5	93.5	Wide σ
F5	0.0	0.5	93.0	Narrow σ
F6	+0.05	1.5	92.5	Right shift + Wide σ
F7	−0.05	0.5	92.0	Left shift + Narrow σ

**Table 7:** Ablation study on fuzzy inference and layered fusion.

Method	Layer-Wise Fusion	Fuzzy Inference	Accuracy (%)
Global-Fuzzy	×	√	92.5
Layered-Avg	√	×	88.5
FedFZ	√	√	**95.5**

*Note:* √ indicates that the component is used, while × indicates that the component is removed. Bold values indicate the best performance among all compared methods.

**Table 8:** Ablation study on layer-type-specific fusion strategy.

Method	Conv Layers	FC Layers	Accuracy (%)
Global	Global	Global	88.5
Layered-Uniform	Uniform	Uniform	94.5
Layered-Heterogeneous	Uniform	Personalized	**95.5**

*Note:* Bold values indicate the best performance among all compared methods.

## Data Availability

Data sharing not applicable to this article as no datasets were generated or analyzed during the current study.

## Abbreviations

The following abbreviations are used in this manuscript:

FedFZ	Fuzzy Rule-Based Federated Layer-wise Fusion
T-S	Takagi–Sugeno
FL	Federated Learning
